# Determination of Average Coulombic Efficiency for Rechargeable Magnesium Metal Anodes in Prospective Electrolyte Solutions

**DOI:** 10.1021/acsami.2c08008

**Published:** 2022-06-28

**Authors:** Ran Attias, Ben Dlugatch, Omer Blumen, Keren Shwartsman, Michal Salama, Netanel Shpigel, Daniel Sharon

**Affiliations:** † Institute of Chemistry and the Center for Nanoscience and Nanotechnology, The Hebrew University of Jerusalem, Jerusalem 919040, Israel; ‡ Department of Chemistry and BINA—BIU Center for Nanotechnology and Advanced Materials, Bar-Ilan University, Ramat-Gan 5290002, Israel

**Keywords:** Mg batteries, metal anode, coulombic efficiency, electrolyte solutions, electrodeposition

## Abstract

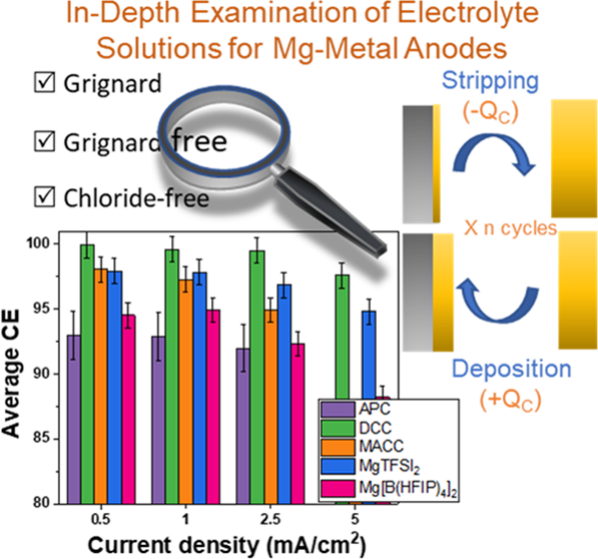

The design of electrolyte solutions that permit reversible and efficient Mg metal electrodeposition is one of the most important tasks in the development of rechargeable Mg batteries. Several types of electrolyte solutions for Mg metal anodes have been developed and explored over the last two decades. These investigations have contributed to a better understanding of the Mg deposition and stripping processes. However, the Coulombic efficiency (CE) for reversible electrodeposition reported for these various systems and their performance in comparison to one another remained unclear. We used rigorous electrochemical methods to accurately quantify the average CE of the major electrolyte solutions considered for secondary Mg metal batteries. We demonstrated how changes in the experiential protocols influence CE measurements, resulting in inconsistent reports. Even though exceptional efficiency has been reported for a variety of systems, we discovered that the only candidate that currently meets the 99% CE benchmark during a prolonged cycling procedure is the dichloro-complex, which is a first-generation Grignard-based electrolyte solution. Second- and third-generation Grignard-free and chloride-free solutions showed reasonable CE only when the deposition currents densities were lowered. This comprehensive and systematic investigation will help to create a more accurate treasure map for potential electrolyte solutions for rechargeable Mg metal anodes.

## Introduction

Metallic anodes for rechargeable batteries offer higher gravimetric and volumetric energy density than currently available intercalation-based anodes. Due to their low reduction potentials and high energy density, lithium (Li),^1^ sodium (Na),^2^ and magnesium (Mg)^3^ have attracted the greatest attention in nonaqueous rechargeable metal batteries. Mg has several significant advantages over Na and Li, including a higher volumetric capacity (3833 vs 2046 mAh/cm^3^ and 1136 mAh/cm^3^ for Li and Na respectively) and a lower reduction potential (−2.37 V vs standard hydrogen electrode (SHE) vs −3.04 V for Li and −2.71 V for Na).^4,5^ Furthermore, considering the recent surge in Li demand and cost, the availability and low cost of Mg metal make Mg-metal-based batteries particularly appealing.

Despite all of the benefits listed and extensive research over the last two decades, the electrochemical performance of Mg metal anodes remains unsatisfactory. One of the primary reasons for this is that Mg has a strong tendency to form passivating surface coatings in a wide range of electrolyte solutions.^6^ As a result, Mg deposition and stripping processes can be very inefficient, as seen by the low efficiency and short cycle life of Mg anodes. Li metal, on the other hand, generates a passivating layer that allows diffusion of Li ions to the active metal sites.^7,8^ As a result, despite their great volumetric capacity, Mg anodes are currently less attractive than other metal-based batteries due to their inefficient electrodeposition and dissolution processes.

Over the last two decades, significant efforts have gone into developing electrolyte solutions capable of reversibly depositing Mg metal.^9−11^ Some of the first proposed electrolyte solution systems were based on ethereal mixtures of organometallic and Mg salt compounds.^12,13^ The goals of keeping the Mg metal unpassivated as well as making electrolyte solutions show outstanding cathodic stability were the driving forces for this choice. Attempts have been undertaken in recent years to move to a simpler and safer electrolyte solution.^14−16^ One of the primary reasons for turning to these solutions is that they may provide improved oxidative stability, which can be crucial for battery components such as current collectors and cathode materials. It is acknowledged, however, that increased oxidative stability cannot be attained at the expense of the Mg anode performance. As a result, most investigations of electrolyte solutions for Mg-metal-based systems evaluate the performance of the Mg metal anodes, which is often described by their Coulombic efficiency (CE). Nonetheless, for a number of reasons that will be described in the following paragraphs, there is ambiguity regarding how the various electrolyte solution candidates for Mg metal anodes compare to one another.

In a recent comprehensive study, Adams et al. used different protocols for assessing the CE for the reversible Li electrodeposition process.^17^ They demonstrated that the values obtained for CE can be influenced by a variety of experimental conditions as well as the applied electrochemical technique. It was implied that intentional and unintentional variations in the condition and experimental procedures are the primary causes of inconsistency in reported CE values. Similarly, CE evaluation reports for reversible Mg metal electrodeposition are riddled with ambiguities. We remark that in the last 5 years, rigorous investigations on the CE of Li electrodeposition have been a significant reason for the rapid development of Li-metal-based batteries. Similarly, additional research into precisely evaluating CE of Mg electrodeposition would promote the concept of Mg-metal-based batteries.

Compared to research on reversible Li electrodeposition, assessing reversible Mg electrodeposition can be more challenging. While the leading candidate electrolyte solutions in Li-metal batteries are widely accepted, there is disagreement on which electrolyte solution candidates are ideal for reversible Mg electrodeposition.^6,10^ Furthermore, in many magnesium electrolyte solutions, the formation of electrochemically active ionic complexes complicates the analysis of the electrodeposition and stripping processes. Another factor to consider is that some Mg electrolyte solutions require an electrochemical conditioning treatment before they can perform reversible Mg electrodeposition. Finally, because even trace amounts of environmental contamination and synthetic byproducts in the electrolyte solution can quickly react with the Mg metal,^18^ failing to examine the various solutions under the same rigorous experimental conditions can result in inconsistent analysis and contradictory reports on the obtained efficiency.

In this study, we will thoroughly evaluate and compare the average CE of the following Mg-based electrolyte solutions: (1) Grignard, (2) Grignard-free, and (3) chloride-free. These three electrolyte solution families represent the most promising candidates for secondary Mg metal batteries, as well as the trends and advancements in this field over the last two decades. We use a consistent macrocycling electrochemical procedure to accurately quantify the average CE of the different electrolyte solution systems. The impact of varying current density parameters and depth of charge on the efficiency and shape of Mg metal deposits is also being investigated. According to the findings of this comprehensive investigation, prolonged and effective reversible Mg deposition (i.e. CE ≥ 99%) is still limited to first-generation Grignard-based electrolyte solutions. This highlights the importance of subjecting new electrolyte solutions to rigorous and demanding testing protocols to effectively expose their potential flaws. These useful procedures and data will help researchers improve present and future electrolyte solutions for rechargeable Mg metal anodes.

## Results and Discussion

### Protocol for Assessing Reversible Mg Electrodeposition

Coulombic efficiency is the most used parameter for describing the degree of reversibility of electrochemical processes. For practical rechargeable metal batteries that can withstand hundreds of stable charge–discharge steps, Coulombic efficiency must be greater than 99%.^19^ It is important to remember that the CE is influenced by the electrochemical process and measurement settings rather than being an intrinsic property of the electrochemical system itself.^17^ Hence, it is critical to use precise and systematic electrochemical processes and conditions when comparing the Coulombic efficiency of various types of Mg-based electrolyte solutions. The cyclic voltammetry (CV) method is frequently used to calculate the CE of Mg electrodeposition process. However, CV may be an inappropriate measuring technique for CE assessment due to the sluggish kinetics of some electrochemical processes, which may distort the voltammetric response due to the influence of the so-called IR drop. Furthermore, the variations in current density and the small amount of cycled Mg metal used in CV experiments do not accurately reflect actual Mg cell operation. As a result, galvanostatic cycling is more precise in estimating the practical CE of the cell than repeated CV sequences.

**Figure 1:**
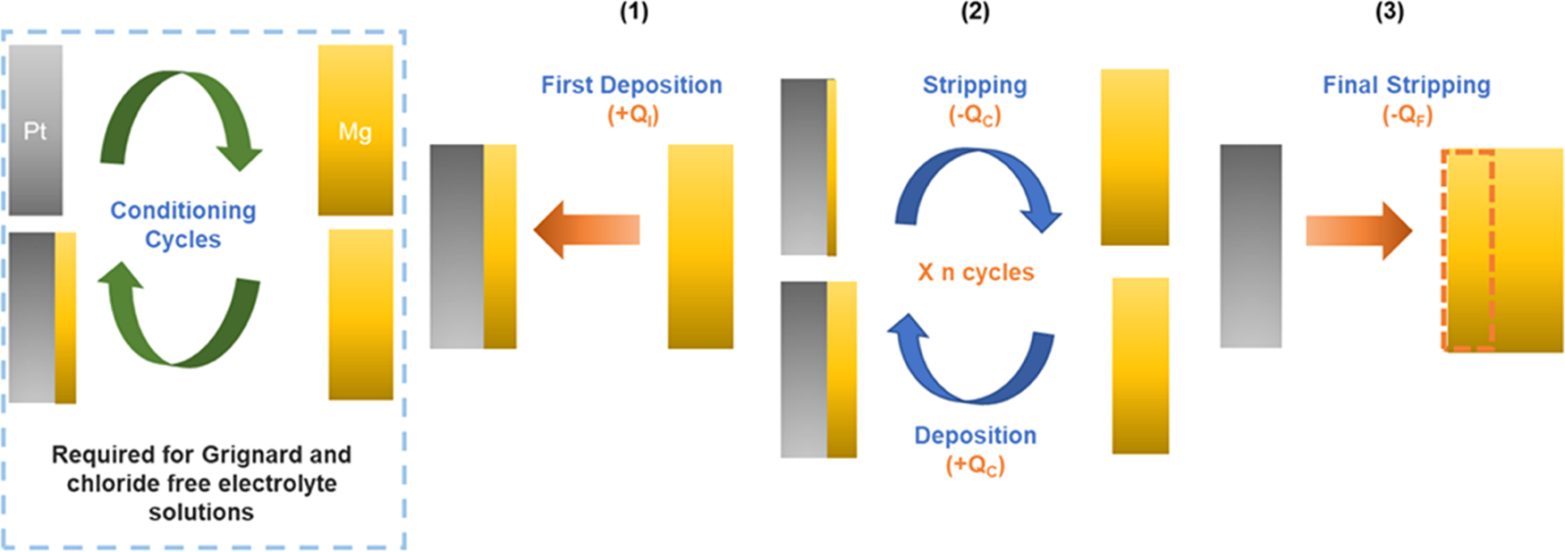
Illustration of the macrocycling protocol for the evaluation of average Coulombic efficiency for reversible electrodeposition of Mg metal.

The galvanostatic “reservoir” method, or “macrocycling” procedure, proposed by Aurbach et al. for determining the average CE of electrodeposition processes, has recently resurfaced as a useful tool for evaluating Li and Zn metal anodes.^17,20−22^Figure 1 depicts the galvanostatic macrocycling sequence for determining the average CE of reversible Mg electrodeposition. The electrochemical cell consists of “inert” metal as a working electrode (WE) (such as Pt or Ni) and a reactive metal (Mg) counter electrode (CE). The procedure starts with a galvanostatic step where a reservoir of Mg (*Q*_I_) is electrodeposited on the bare metal electrode (in this study, a Pt electrode was used). Rather than stripping and depositing all of the Mg (*Q*_I_) in each cycle, only a portion of the reservoir denoted as *Q*_C_ (*Q*_C_ < *Q*_I_) is cycled for a predetermined number of cycles. The test is ended with a final stripping procedure to remove all of the plated Mg from the Pt substrate; the amount of Mg removed during this stage is referred to as *Q*_F_. It is worth noting that a cutoff voltage of ±1 V vs Mg/Mg^2+^ is used as a boundary for both the deposition and stripping process.

Except for Grignard-based solutions, the macrocycling sequence must be done after the examined electrolyte solution has been subjected to an electrochemical conditioning treatment. Although the conditioning process can differ between reports, the overall concept remains the same. Galvanostatic cycling of a cell identical to that used in the macrocycling procedure, which includes the desired electrolyte solution, is the main part of the protocol. Because the electrolyte solution has not been conditioned and Mg passivation is unavoidable, Mg deposition/stripping is inefficient at first and requires a large overpotential to work. The freshly deposited Mg reacts with impurities in the solution during the conditioning process, which is the primary cause of Mg passivation and deactivation.^23^ Other electrolyte solution reactions that produced active Mg ionic species are also thought to be capable of preparing the solution for reversible Mg deposition.^24^ Regardless, once the deposition/dissolution process has stabilized, the electrolyte solution is ready to use in a new cell for the macrocycling test.

The macrocycling method might result in one of two outcomes, both of which will affect how we calculate the average CE. In the first scenario, the procedure went as planned, and the cell did not reach the cutoff voltage during the long cycling procedure. In this instance, we will calculate the average CE using eq 1
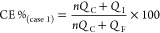
1which considers *Q*_C_ as well as the difference between *Q*_I_ and *Q*_F_. When *Q*_I_ = *Q*_F_, the average CE is 100% because no Mg metal was lost between the initial deposition (*Q*_I_) and final stripping (*Q*_F_) processes. However, because some of the Mg deposited during the initial process cannot be recovered, *Q*_F_ is usually smaller than *Q*_I_. One reason for this inefficiency is that the charge transfer during the macrocycling procedure may have been consumed by an irreversible electrochemical parasitic reaction instead of reversible Mg electrodeposition.^25−27^ Alternatively, the charge transfer might have resulted in Mg deposition, but the freshly deposited Mg was permanently lost owing to mechanical (peeling) or chemical (corrosion) side reactions.^18,28−31^ As a result, in addition to the difference between *Q*_I_ and *Q*_F_, we include the number of cycles and the amount of Mg that was deposited/striped during the long cycling procedure when calculating the average CE. This is because the influence of parasitic electrochemical, physical, and chemical side reactions becomes increasingly significant as the number of cycles and amount of Mg deposited is increased.

The second scenario is that the macrocycling process is terminated prematurely because the cutoff voltage is reached before the extended deposition and stripping cycles are completed. In this case, the final stripping (*Q*_F_) stage is not reached, and the average CE can be described using the following simplified eq 2
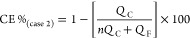
2The average CE in this case is defined by the amount of cycled Mg and the number of cycles performed prior to the cell reaching the cutoff voltage.

In this study, the average CE of different classes of Mg-based electrolyte solutions are thoroughly evaluated and compared. To test these solutions, the macrocycling parameters used were an initial deposition of *Q*_I_ = 2.88 C, followed by 100 cycles of stripping and deposition of *Q*_C_ = 0.25·*Q*_I_ before the final stripping step (*Q*_F_). These measurements were carried out at four different current densities: 0.5, 1, 2.5, and 5 mA/cm^2^. If under these conditions, the evaluated electrolyte solution system did not complete 100 cycles before reaching the cutoff voltage, and the amount of cycled Mg was reduced to *Q*_C_ = 0.05·*Q*_I_.

#### Grignard-Based Electrolyte Solutions

Electrolyte solutions based on Grignard regent were the first to show efficient reversible electrodeposition of Mg metal in a full battery configuration.^13^ The absence of passivation layer development on the Mg surface is associated with the ability of Grignard-based solutions to reversibly deposit Mg. We will examine two members of this family the dichloro-complex (DCC) and all-phenyl complex (APC) solutions which are the product of reactions between organo-magnesium and organic halo aluminum compounds in ethereal solvents.

##### Dichloro-complex DCC

**Figure 2:**
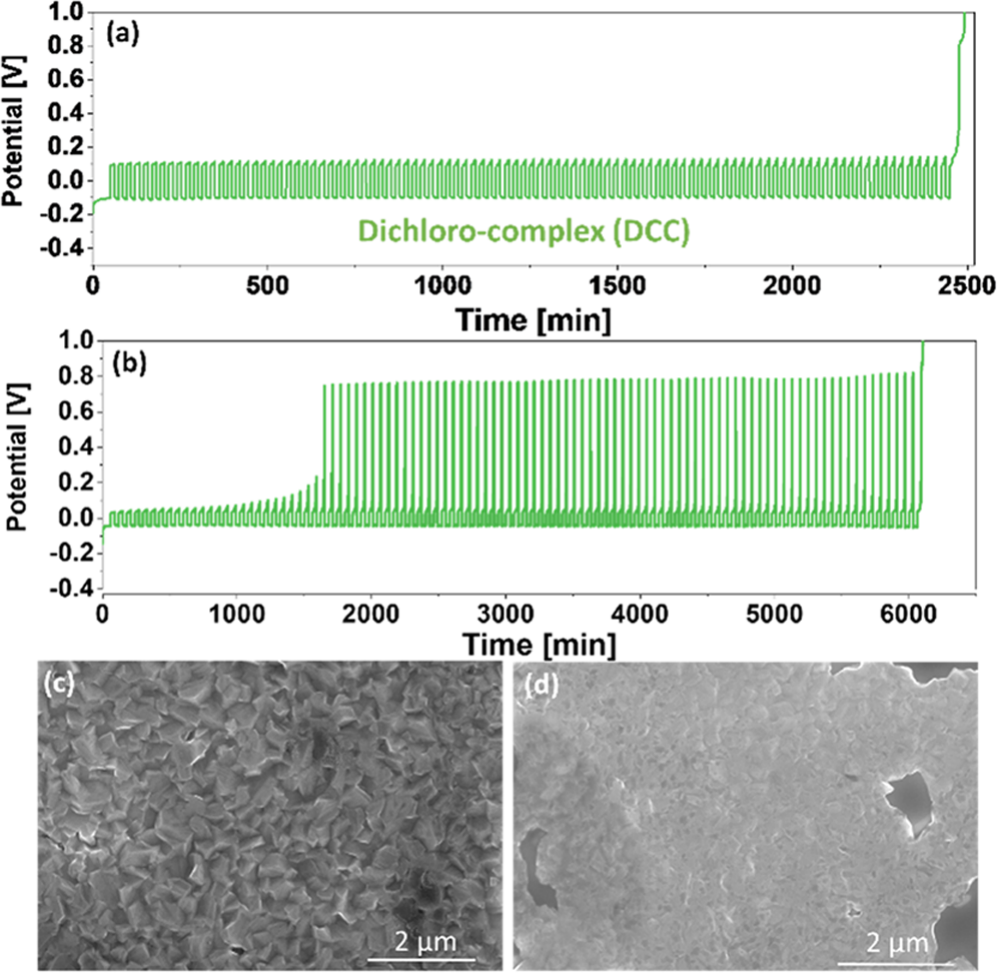
(a) Macrocycling measurements for DCC solutions at 25% depths of discharge at 1 mA/cm^2^, where Pt is used as WE and Mg foils as both CE and reference electrode (RE). (b) Macrocycling measurements for DCC solutions at 50% depths of discharge at 0.5 mA/cm^2^. Scanning electron microscopy (SEM) image shows a Pt electrode after the first Mg electrodeposition process in the DCC solution at a current density of (c) 1 mA/cm^2^ and (d) 5 mA/cm^2^.

The first examined formulation is the 1:2 Bu_2_Mg/EtAlCl_2_ in tetrahydrofuran (THF), which is also known as dichloro-complex (DCC).^12,32^ A representative voltage profile of the macrocycling procedure in DCC solution at a current density of 1 mA/cm^2^ is presented in Figure 2a. The final stripping process (*Q*_F_) yielded a charge of 2.58 C, indicating that 0.30 C of Mg was lost during 100 cycles, and thus the calculated average CE is equal to 99.59% using eq 1. The average CE lowers as the current density rises in the DCC instance, as it does in the other electrolyte solutions, implying that Mg loss during cycling is larger at higher current densities, even if the depth of discharge is not changed. The decline in efficiency can be caused by a variety of irreversible electrochemical, chemical, and physical reactions. The exact reaction will be discussed briefly later in the paper; however, the main goal of this study is to provide an accurate description of the reversible Mg electrodeposition efficiency performance for the various prospective Mg-based electrolyte solutions, rather than to suggest all of the possible side reactions that can occur in these complex systems. Nonetheless, the DCC system drops from the 99% CE benchmark only at a current density of 5 mA/cm^2^ (97.60%), which is regarded as very high for metal anodes in nonaqueous electrolyte solutions.

To evaluate how the DCC electrolyte solution performs under even more demanding conditions, we increase the amount of cycled Mg to be *Q*_C_ = 0.50·*Q*_I_ (Figure 2b). Even under these settings, the cell completed 100 cycles, and the calculated CE only decreased to 99.38%. Nonetheless, we can observe that the stripping potential gradually increased to 0.8 V during the cycling sequence. This rise in striping overpotential might be attributed to the formation of a more resistant Mg metal layer during the deep electrodeposition procedure or to limited Mg transport in the solution. Yet, exhibiting CE of above 99% with a discharge depth of 50% is considered exceptional for any metallic anode. Previous study on DCC electrolyte solution reported that the CE for Mg electrodeposition was significantly lower than 100%, especially in the early cycles.^33^ It was proposed that the main cause of the low efficiency could be electrolyte solution decomposition.^33,34^ It is worth noting that in these studies potentiostatic and potentiodynamic (CV) measurements were used to assess the CE. Measuring charge transfer by applying a potential to an electrode can result in unwanted side reactions that do not occur when measuring by galvanostatic methods. Furthermore, completely removing the Mg from the Pt/Au substrate can enhance possible substrate effects, particularly in early cycles. More analysis of the DCC solution, however, is required to determine whether and to what extent electrolyte solution decomposition occurs during cycling. We can conclude that electron transfer at the electrode surface is efficient because the deposition overpotential did not increase during cycling. That is, even if surface side reactions occurred with the DCC solution, the resulting layer is either conductive or thin enough to allow efficient Mg deposition.

High-resolution (HR) SEM imaging was utilized to determine whether there is a correlation between CE and deposit morphology, as well as to validate the development of Mg metal crystals during electrodeposition at different current densities. To compare the samples, a Mg deposit thickness of 1 μm (estimated from the transferred charge) was electrodeposited using a galvanostatic step with varied current densities. At all current densities, we can see that homogeneous Mg deposits with hexagonal crystallite shapes cover the Pt electrode (Figure 2c,d). The crystallite size reduces with increasing current density, implying that the limiting factor of the electrodeposition process at high current may be the lateral diffusion of Mg ions on the electrode surface until they reach energetically favorable sites. The increased surface area at high current density may intensify potential side reactions during cell cycling, resulting in an apparent drop in CE. Additionally, physical detachment of small and more fragile crystallites from the anode surface might result in Mg loss. However, these observations are insufficient on their own to support this hypothesis, and more extensive research is required to quantify possible irreversible reactions.

##### All-Phenyl Complex (APC)

While DCC supports reversible Mg electrodeposition, its anodic stability (≈2.2 V vs Mg^0^/Mg^2+^) limits the selection of suitable cathode materials that can be used in conjunction with Mg metal anodes.^35^ It was suggested that removing all β-located hydrogen from the DCC can significantly increase the solution oxidation stability.^36^ To do so, the all-phenyl complex (APC) electrolyte solution in ethereal solvent from the reaction products PhMgCl and AlCl_3_ was synthesized.^37,38^ APC was shown to have good anodic stability (3.3 V vs Mg^0^/Mg^2+^) while maintaining a low Mg deposition and stripping overpotential. The current study is primarily concerned with the high CE values reported for recurrent Mg deposition in APC solution. These values were obtained using cyclic voltammetry (CV) micro cycling methods, which, as previously stated, are not optimal for reliable CE evaluation.^36,37,39,40^ In the following sections, the above-mentioned approach will be utilized to assess the APC solution’s long-term Mg reversible electrodeposition efficiency.

**Figure 3:**
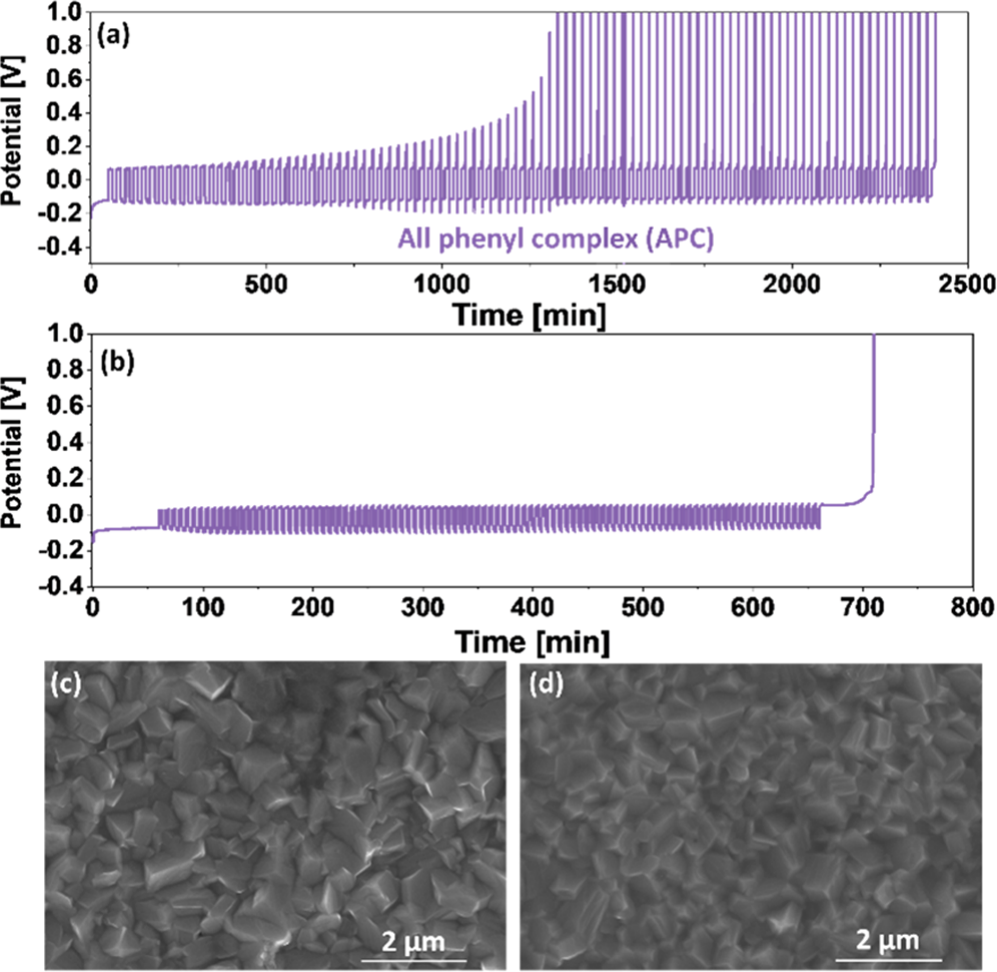
(a) Macrocycling measurements for 2 M APC/THF solutions at 25% depths of discharge at 1 mA/cm^2^, where Pt is used as WE and Mg foils as both CE and RE. (b) Macrocycling measurements for APC solutions at 5% depths of discharge at 0.5 mA/cm^2^. SEM image shows a Pt electrode after the first Mg electrodeposition process in APC solution at (c) 1 mA/cm^2^ and (d) 5 mA/cm^2^.

Figure 3a depicts a representative macrocycling voltage profile for cell with 2 M APC/THF solution at 1 mA/cm^2^. The cell clearly exceeds the cutoff voltages early in the macrocycling process, before the 100 cycles are completed. Using eq 2 for prematurely failing cells, we determined that the average CE is 92.85%. Even at a lower current density of 0.5 mA/cm^2^, the macrocycling process reaches the cutoff voltage after 52 cycles, resulting in an average CE of 92.98%. To see if the average CE would improve with less demanding macrocycling settings, we reduced the amount of cycled Mg by 5 times (*Q*_C_ = 0.05·*Q*_I_). The voltage profile in Figure 3b shows that the cell successfully completed the macrocycling procedure under these conditions. Despite this, the calculated efficiency (CE = 97.13%) is much lower than that of the DCC electrolyte solution.

We note that it is thought that efficient reversible Mg deposition within the Grignard-based electrolyte solutions relates to the reductive nature of the organometallic components. The organometallic species act as scavengers, removing contaminants such as water, oxygen, carbon dioxide, and other protic residues (from synthesis) that would otherwise passivate metallic Mg. While APC is an organometallic-based electrolyte solution, it does not have the same characteristics as DCC in terms of reversible Mg deposition. These findings imply that, while consumption of contaminations and side reactants is important for Mg electrochemistry, it is not a guarantee of high average CE.

Aside from chemical similarities, the morphology of electrodeposited Mg in APC solutions as a function of current densities is also very similar to that observed in DCC solutions (Figure 3c,d), raising the question of what is causing the major difference in average CE between these two electrolyte solutions. According to Pan et al., adding MgCl_2_ salt to the APC solution increased its reversible Mg electrodeposition efficiency.^39^ They proposed that the presence of additional chloride anions promotes the formation of surface-active Mg–Cl ionic complexes, which improves the APC solution’s ability to deposit and strip Mg. This could imply that these or other types of active species are naturally produced in DCC solutions, resulting in greater efficiency. It has also been proposed that spontaneous galvanic reactions between aluminum-based anionic complexes in the APC solution and the Mg anode can lead to stripping of Mg metal and deposition of Al.^28,41^ Possible side reactions of the APC solutions with the electrode surface could be inferred from the macrocycling deposition step, where the current spike could imply that the system is breaking through a barrier layer to initiate the deposition process. Due to the difficulty in isolating and characterizing the active ionic complexes in these systems, the core difference between the different Lewis acid-derived Mg-based electrolytes is still not fully understood. While the APC outperforms DCC solution in terms of oxidation stability, its reversible Mg deposition behavior needs to be improved.

#### Grignard-Free Electrolyte Solutions

##### Magnesium Aluminate Chloride Complex (MACC)-Based Solutions

Because of the strong chemical reactivity of Grignard-based electrolyte solutions and compatibility with cathode materials, and current collectors, it was proposed that removing the Grignard regents from the solutions could help to alleviate this issue. Doe et al. propose that a 2:1 ratio of MgCl_2_ and AlCl_3_ reactions in THF solvent can produce an electrolyte solution containing reactive Mg ionic species, which they termed magnesium aluminate chloride complex (MACC).^42^ Due to the lack of an organometallic contaminate scavenger in the MACC solution, it should be preconditioned before use in Mg metal systems. The conditioning process, which consists of extended galvanostatic cycling of the cell, is intended to aid in the elimination of electrolyte solution impurities. It is also proposed that the conditioning process can cause changes in active Mg complexes and the formation of free chlorides species, both of which can improve Mg electrodeposition.^24^ While the exact conditioning mechanism is still unclear, for our purpose, it is important to note that in certain solutions, a conditioning step must be applied prior to the macrocycling procedure.

**Figure 4:**
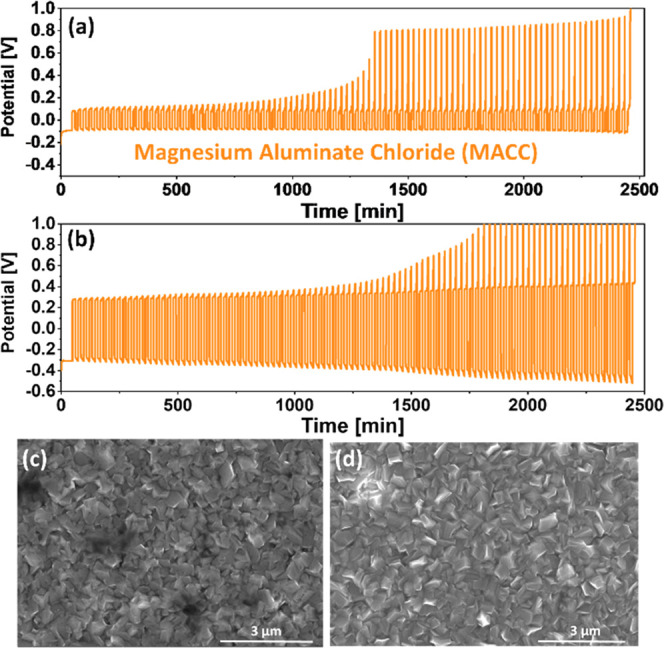
(a) Macrocycling measurements for conditioned MACC/THF solutions at 25% depths of discharge where Pt is used as WE and Mg foils as both CE and RE (a) at 1 mA/cm^2^ and (b) at 2.5 mA/cm^2^. SEM image shows a Pt electrode after the first Mg electrodeposition process in conditioned MACC solutions at (c) 1 mA/cm^2^ and (d) 5 mA/cm^2^.

To measure the average CE of the MACC, we applied the macrocycling procedure on a conditioned electrolyte solution. After the conditioning procedure, both the Pt and Mg electrodes must be replaced for the macrocycling sequence to obtain precise and consistent average CE values. Figure 4a shows a representative voltage profile of the macrocycling procedure at 1 mA/cm^2^. The macrocycling procedure completed the 100-cycle sequence without reaching the cutoff voltages, and the computed average CE is 97.27%. Even though the cell has completed 100 cycles, we can observe that the stripping potential is progressively rising toward the cutoff voltage. Hence, we should anticipate the MACC-based cell to reach the cutoff voltage (1 V) quickly by increasing the electrochemical parameters. This is exactly what happened when we increased the current density to 2.5 mA/cm^2^ (Figure 4b), as the cell failed to complete the cycling operation after 75 cycles (CE = 94.93%). It is worth noting that as current density increases, so does the deposition overpotential, as well as the stripping overpotential. SEM imaging was used to examine the deposition products of MACC-based cells at various current densities (Figure 4c,d). One can see that when the current density increases from 1 to 5 mA/cm^2^, the deposited Mg layer becomes less planar. This could explain the increase in potential (Figure 4b) caused by more parasitic reactions and passivation of the Mg surface due to its larger surface area. Diverse side reactions with the electrolyte and impurities in MACC solution are linked with irreversible Mg deposition behavior, but the specific mechanism is still uncertain due to the conditioning process and the production of various ionic complexes that can participate in these reactions. Another concern with MACC solutions is their lack of long-term cycling stability. When the MACC solution is left in the cell for an extended period of time, another conditioning step is often required prior to cycling. Furthermore, while MACC is free of Grignard reagents and thus considered safer, the presence of MgCl_2_ and AlCl_3_ in the solution causes an increase in unwanted corrosive reaction, which can lead to decreased efficiency and increased potential over time.

Next, we wanted to explore if we could increase the CE performance of MACC-based cells by lowering the amount of cycled Mg during the macrocycling process, like the APC electrolyte solution (Figure S6). As expected, when the depth of discharge was reduced to 5%, the average CE (98.33%) increased with respect to the cells that discharged to a plating depth of 25% (98.07%). Furthermore, during the recurring deposition and dissolution processes, no substantial overpotential was developed, showing that the cell is not on the verge of failing. The results show that the degree of reversibility for Mg deposition in MACC-based solutions is highly dependent on the electrochemical procedure parameters.

##### Electrolyte Solutions Based on MgTFSI_2_ Salt

Using simple Mg salts that do not contain organometallic compounds or chloride anions is one method for suppressing corrosion reactions, improving safety, and extending anodic stability of the Mg-based solution. Among the various options, electrolytes based on the bis(trifluoromethanesulfonyl)imide (TFSI) anion are among the most promising candidates.^15,43^ Pure MgTFSI_2_-based solutions were eventually discovered to be unsuitable for efficient reversible magnesium deposition; however, the addition of chloride salts and a conditioning step improved their performance.^23,44,45^ In this study, we will determine the average CE of a solution containing MgCl_2_ and MgTFSI_2_ in a 2:1 ratio in 1,2-dimethoxyethane (DME) solvent. As with all Grignard-free solutions, the as-prepared MgTFSI_2_-based solution requires conditioning before it can be deployed for the macrocycling procedure.

**Figure 5:**
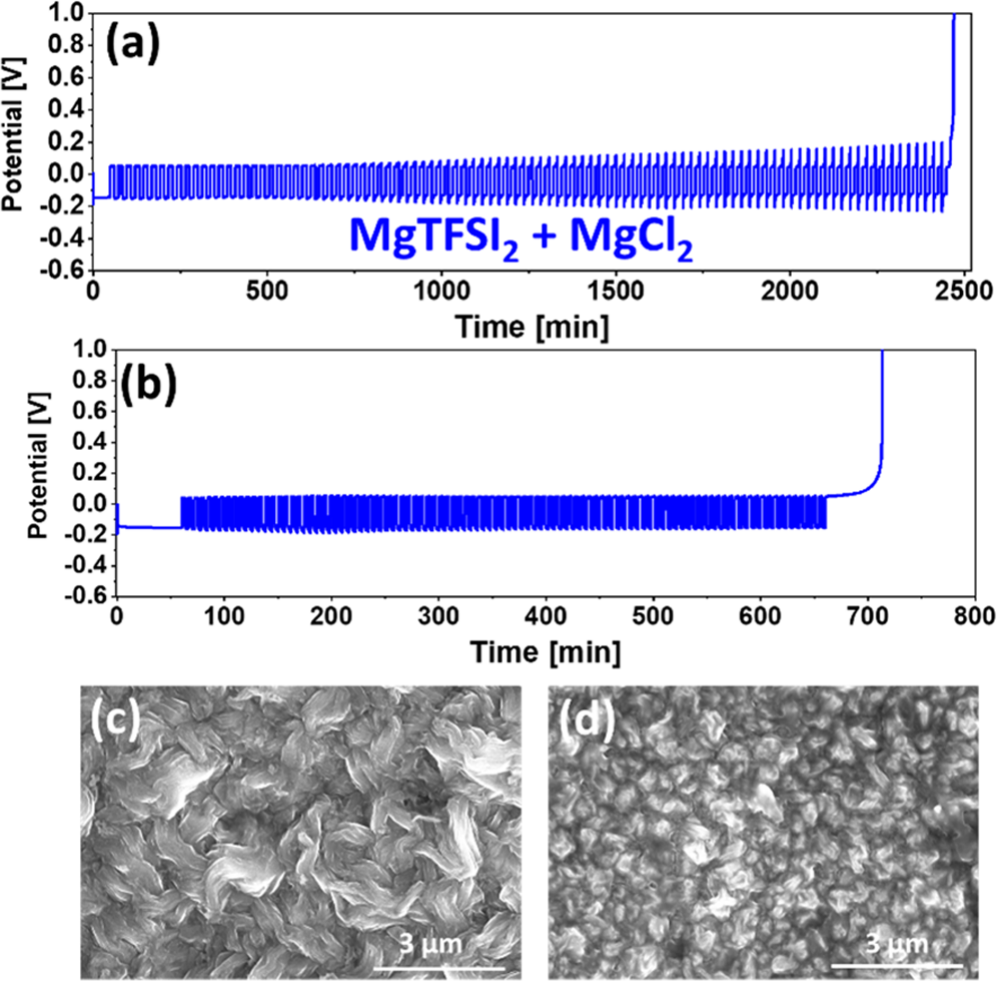
(a) Macrocycling measurements for conditioned MgTFSI_2_–MgCl_2_/DME solutions at 25% depths of discharge at 1 mA/cm^2^, where Pt is used as WE and Mg foils as both CE and RE. (b) Macrocycling measurements for conditioned MgTFSI_2_-based solutions at 5% depths of discharge at 0.5 mA/cm^2^. SEM image shows a Pt electrode after the first Mg electrodeposition process in conditioned MgTFSI_2_-based solutions at (c) 1 mA/cm^2^ and (d) 5 mA/cm^2^.

A representative macrocycling voltage profile of the 1 mA/cm^2^ cell is presented in Figure 5a. We can observe that the cell completed 100 cycles and presented an average CE of 97.83%. During the cycling, the cell containing MgTFSI_2_ solution exhibits a progressive increase in the overpotential in a similar fashion that we observed for MACC-based electrolytes. However, in contrast to the MACC solution-based cells where an increased polarization was observed only upon the stripping cycles, a symmetrical increase in the overpotentials in both plating and striping processes was observed for the MgTFSI_2_ solution-based cells. The symmetric increase in the overpotential may indicate the formation of a metastable layer on the plated Mg anode. The gradual increase in the overpotential could imply that the layer accumulates on the Mg surface throughout the cycling step, increasing its resistance. It is thought that the formation of this layer is caused by surface reactions between the Mg metal and the TFSI anion during the deposition process.^23,26,46^ Nonetheless, we can see that even with the increased potential, the cell was able to present a reasonable CE under these electrochemical conditions.

The morphology of the deposited Mg is another distinctive feature of TFSI-based solutions. As shown in Figure 5c,d, the morphology of the deposited film is made up of interweaving large bundles and some smaller crystallites of Mg metal. This type of complex morphology is notably different from the sharped edge crystals formed in the other electrolyte solution systems investigated here.^46,47^ Similar filamentary metal growth has been reported in Li-metal systems, and it has been proposed that the presence of passivation film might contribute to the production of these types of morphologies.^48^ In general, passivation film generated in Mg cells is expected to deactivate the anode because Mg-based inorganic and organic compounds are poor Mg^2+^ conductors. Having said that, it is possible that some degree of Mg passivation, such as that seen in MgTFSI_2_-based solutions, can still enable Mg deposition and dissolution process.^23,46,49−51^

The effect of possible irreversible reactions and surface layer formation is even more at higher current densities of 2.5 and 5 mA/cm^2^ (Figure S4). In these cases, the cells failed to complete 100 cycles and the calculated CE fell to 95.83 and 94.80%, respectively. Electrolyte solution side reactions or the electrolyte solutions limited ionic conductivity might explain the behavior at higher current densities. The ionic conductivity argument is less likely because of the high concentration of chloride anions and the relatively strong disassociation of TFSI anions,^32,45^ whereas the documented degradation of TFSI anions makes the formation of a resistive layer more likely.^26,27^

Finally, we looked at how much the average CE might be improved by lowering the macrocycle settings. Figure 5b shows the macrocycling voltage curve of a MgTFSI_2_-based solution with *Q*_C_ = 0.05·*Q*_I_. In comparison to the 97.91% estimated for *Q*_C_ = 0.25·*Q*_I_, the CE rises to 98.16%. Although the MgTFSI_2_-based solution did not meet the 99% CE criterion, they appear to behave consistently at all current densities tested, and its performance is only inferior to that of the DCC electrolyte solution.

#### Chloride-Free Electrolyte Solutions

All of the electrolyte solutions discussed above included chloride ions, which are corrosive to the relevant current collectors and metal oxide cathodes employed in Mg battery systems. As a result, a chloride-free electrolyte solution for Mg metal batteries is much sought after. The difficulty in identifying such a system is the critical role of chloride ions in the electrodeposition and dissolution process of Mg metal. It is proposed that in solutions containing chloride ions, [Mg – Cl]^+^ ionic complexes can adsorb to the anode surface, inhibiting other species such as the solvent molecule from reacting with the Mg metal and passivating its surface.^52^

In recent years, a variety of halide-free solutions have been investigated to demonstrate efficient Mg deposition behavior.^14,53−55^ Fichtner et al. proposed that cycling of Mg metal cells could be accomplished using magnesium tetra kis(hexafluoroisopropyloxy)borate (Mg[B(HFIP)_4_]_2_).^14,56^ According to Dlugatch et al., this solution necessitates a thorough conditioning process to remove reactive contaminants that cause unavoidable passivation and deactivation of the Mg metal.^54^ This is unsurprising given that all Grignard-free electrolyte solutions lack strongly reductive agents capable of scavenging the different contaminants. Furthermore, the absence of chloride ions, which can partially screen the Mg metal surface from undesirable reactions, makes electrolyte solution conditioning even more crucial in comparison to MACC and MgTFSI_2_-based solutions.

**Figure 6:**
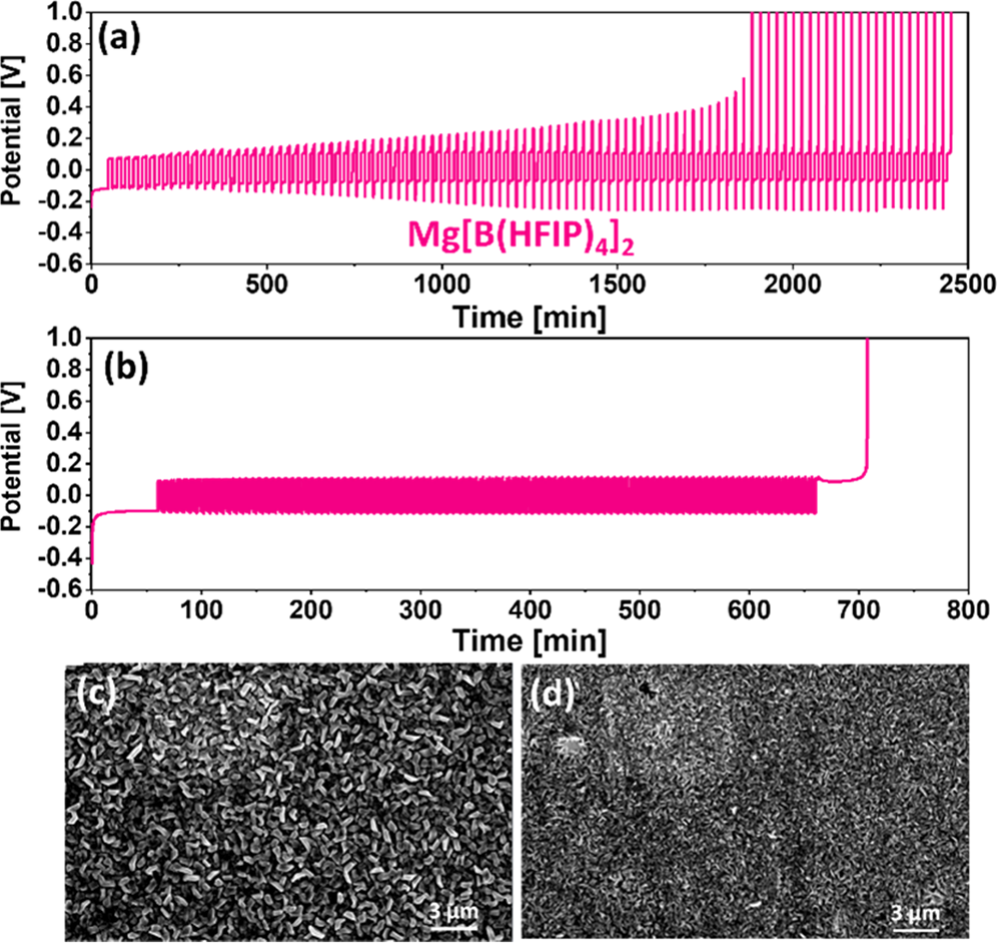
(a) Macrocycling measurements for conditioned Mg[B(HFIP)_4_]_2_/DME solutions at 25% depths of discharge at 1 mA/cm^2^, where Pt is used as WE and Mg foils as both CE and RE. (b) Macrocycling measurements for Mg[B(HFIP)_4_]_2_/DME solutions at 5% depths of discharge at 0.5 mA/cm^2^. SEM image shows a Pt electrode after the first Mg electrodeposition process in conditioned Mg[B(HFIP)_4_]_2_/DME solutions at (c) 1 mA/cm^2^ and (d) 5 mA/cm^2^.

Figure 6a shows a representative macrocycling voltage profile of a conditioned 0.3 M Mg[B(HFIP)_4_]_2_/DME-based cell cycled at a 1 mA/cm^2^ current density. We can see that the cell failed to complete 100 cycles and had an average CE of 94.93%. At higher current densities of 2.5 and 5 mA/cm^2^, the cells failed to complete 100 cycles, and the CE dropped to 93.83 and 92.80%, respectively (Figure S5). The voltage profile shows that the overpotential of both the deposition and stripping processes gradually increases over the cycling sequence. As previously stated, symmetric increases in the overpotential suggest that metastable resistive layer buildup is passivating the Mg metal, generating gradual increases in the overpotential. The fact that both chloride-free Mg[B(HFIP)_4_]_2_ and low-chloride-concentration MgTFSI_2_-based solutions exhibit this behavior suggests that the shortage of active chloride ions in the solution significantly increases the reductive behavior of the Mg metal toward the electrolyte solution, resulting in the accumulation of side products that passivate the anode surface. It should be noted that cells fail during the first cycle in unconditioned Mg[B(HFIP)_4_]_2_ solutions, which might imply on the magnitude of possible irreversible side reactions during cell operation. The presence of borate and fluoride species on the Mg metal may indicate that the electrolyte is decomposing;^54^ however, a more thorough analysis is required to completely comprehend the reason for its electrochemical behavior.

The HR-SEM micrographs of electrodes from conditioned Mg[B(HFIP)_4_]_2_-based cells reveal a homogeneous layer of filament-like deposits rather than hexagonal plates (Figure 6c). We can see that the crystallite size decreases with increasing current density, like the other electrolyte solutions, indicating that lateral diffusion of the Mg ions is the limiting factor of the electrodeposition process at high current. (Figure 6d). While it was previously assumed that all nonaqueous electrolyte solutions should exhibit smooth layer-by-layer Mg electrodeposition, the morphology observed in the TFSI and Mg[B(HFIP)_4_]_2_-based solutions suggest the presence of alternative Mg metal growth mechanisms that should be investigated further.^46^

To see if we could improve the average CE at 0.5 mA/cm^2^ current density, we reduced the depth of discharge from 25 to 5% (Figure 6b). The cell completed 100 cycles with no significant increase in overpotential, and the calculated average CE improved to 96.38%, up from 94.52% in a 25% depth of discharge protocol. This demonstrates how electrochemical setting changes can have a significant impact on measured CE results for identical electrolyte solution systems. For example, the impact of irreversible chemical and electrochemical side reactions is less notable in short and shallow deposition/stripping cycles than in long and deep cycles. Nonetheless, these average CE values are highly impressive for Cl-free systems and should not be taken for granted.^53,55^ Furthermore, the increased anodic stability of Cl-free electrolyte solutions makes future efforts to enhance their CE very compelling.

## Discussion

**Figure 7:**
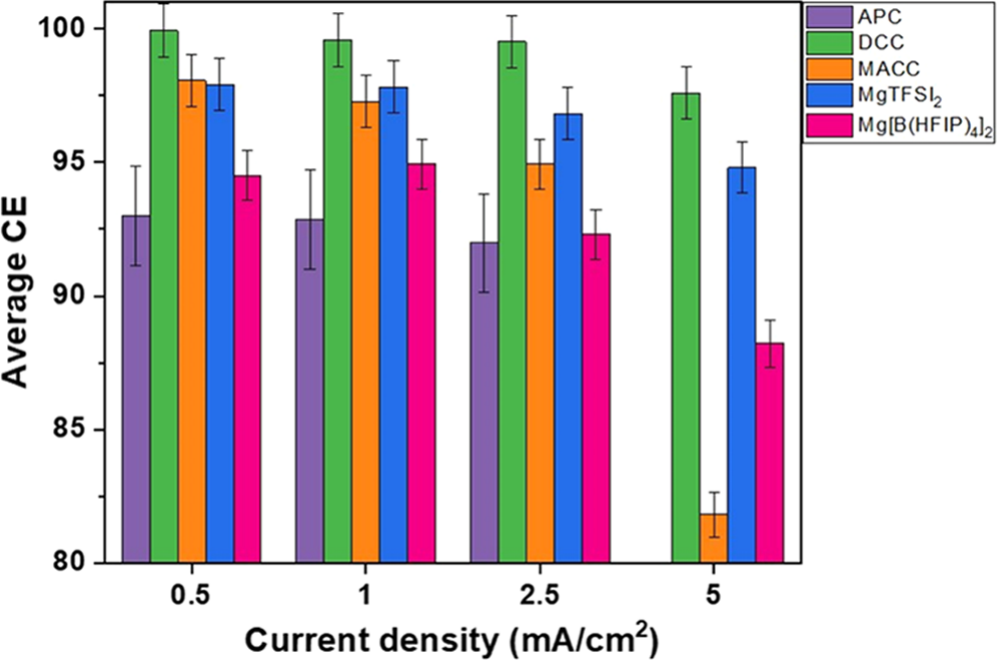
Summary of the calculated average Coluombic efficiency of the different Mg-based electrolyte solutions at different current densities. The error bars represent the standard deviation between different cells. All of the cells were cycled 100 times with a depth of discharge of 25%, which is equal to areal capacities of 0.1, 0.2, 0.5, and 1 mAh/cm^2^ for 0.5, 1, 2.5, and 5 mAh/cm^2^ current densities, respectively.

By examining the different electrolyte systems in the same setup, conditions, and electrochemical procedure, we can honestly compare their degree of reversibility of Mg metal electrodeposition. The graph in Figure 7 presents the calculated average CE of the five-electrolyte solution system at different current densities. One can immediately see that in all current densities, the most efficient system is the DCC. Furthermore, this is the only system that presents efficiency higher than 99%, and even goes to the highly desirable value of 99.9% at low current densities of 0.5 mA/cm^2^. The main proposed reasons for inefficient reversible Mg electrodeposition are irreversible reactions of the Mg metal with impurities or the electrolyte solution, which can also passivate and block the Mg surface. On the other hand, the formation of Mg complexes in solution, particularly Mg–Cl-based complexes, can result in improved reversible Mg deposition. The ability of the Gingered reagent to scavenge impurities as well as its high reduction stability against Mg metal can explain why DCC solution outperforms all other systems. However, due to the limited CE performance of the APC solution, this interpretation is likely not entirely correct. A reactive Grignard reagent capable of “cleaning” the solution appears insufficient for reversible Mg deposition that is both prolonged and effective. It is still uncertain if possible irreversible side reactions of the APC or the formation of less efficient Mg complexes is the reason for the reduced efficiency. The improvement in CE in the APC system when the cycling protocol was considerably shortened could signal that electrolyte solution side reactions were a contributing factor to its inefficiency.

Looking at non-Grignard systems can help us better understand how impurity scavenging, electrolyte solution side reactions, and the formation of active Mg complexes affect the average CE behavior. When used as prepared, these solutions have an average CE of 20–30%. The contaminants are scavenged by electrochemical and chemical processes after the solution has been conditioned, and the average CE improves to over 90%. The scavenging effect can be directly linked to this significant increase in average CE. However, even after a thorough cleaning of the solution, the reversible behavior of the Mg metal during long cycling procedures is limited and does not exceed the 99% CE benchmark. This emphasizes that impurities are the major cause for the inefficiency in these systems, with other possible side reactions with the electrolyte solution, as well as a kinetically inefficient electrodeposition and dissolution process, accounting for less than 10% of the irreversible behavior. This could imply that fine-tuning of these electrolyte solutions could possibly take them past the 99% CE criteria. The addition of Cl^–^ ions can improve the efficiency of deposition and stripping behavior, as evidenced by the impressive CE of the MgTFSI_2_-based solution. The absence of Cl^–^ ions in the Mg[B(HFIP)_4_]_2_-based solution might be one of the reasons for the reduction in the Mg deposition and stripping efficiency. Chloride species adsorbed on the Mg surface are thought to limit the accessibility of other solution species that can irreversibly react with the Mg. However, due to the corrosive nature of chloride, the achievement of a Cl-free solution with reasonable Mg deposition behavior must be acknowledged and appreciated.

## Conclusions

The major electrolyte solution families were characterized under the same conditions and protocols. We found that varying the parameters of the electrochemical procedure for determining the CE can result in considerable changes in the obtained results. Disparities in performance might also be caused by the synthesis, conditions, and treatment of the electrolyte solutions. These variations may lead to discrepancies in the evaluation of the tested electrolyte solution. As a result, future publications should contain experiments in which various parameters (current density, depth of charge, length of the cycle operation, condition protocol, aging, and so on) are investigated to provide a more comprehensive description of the electrolyte solution systems.

The only tested candidate that meets the CE benchmark of ≥99% during prolonged cycling protocol is the DCC electrolyte solution. While it is commonly assumed that its high efficiency is due to the presence of reactive Grignard reagent, the mediocre performance of the other Grignard-based solution, APC, makes this explanation somewhat insufficient. Grignard-free electrolyte solutions show reasonable CE only when the depth of discharge and current densities are minimized. This is particularly notable in Grignard-free solutions, which also exhibit a gradual increase in overpotential throughout the cycling sequence. As more research on Grignard and Grignard-free solutions is conducted, it is necessary to clarify the basic mechanisms and influence of the conditioning process. Is the sole process going on is the consumption of trace contaminants, or are other critical processes taking place? Is the conditioned solution stable at the relevant time scales, or does it need to be conditioned again after some time? Does the mechanism of the conditioning process the same for all Mg-based solutions? Because this process is so important in evaluating potential Mg-based electrolyte solutions, future work should include a thorough investigation of its reaction mechanism as well as optimization of the conditioning protocol. Finally, we demonstrated that with proper conditioning, these solutions may achieve CE of more than 95% under prolonged cycling procedures. Future research should look at whether and how these electrolyte solutions can exceed the 99% criteria. Furthermore, due to the impracticality of the electrochemical conditioning process, alternative conditioning methods with greater commercial viability must be sought. Although we focused on Mg metal anodes in this study, these systematic techniques can and should be applied to other prospective metallic-based battery systems.

## Supporting Information Available

Macrocycling profiles and SEM images and synthesis of all of the electrolyte solution systems (PDF)
