# Supplementary material for: Determination of Average Coulombic Efficiency for Rechargeable Magnesium Metal Anodes in Prospective Electrolyte Solutions
Source: ACS Appl Mater Interfaces. 2022 Jun 28;14(27):30952–61. doi: 10.1021/acsami.2c08008 (PMC9284514; doi:10.1021/acsami.2c08008)
Supplement: Supplementary file 1 — am2c08008_si_001.pdf [file am2c08008_si_001.pdf]

Supporting Information

Determination of Average Coulombic Efficiency for Rechargeable Magnesium Metal Anodes in Prospective Electrolyte Solutions

Ran Attias,[1] Ben Dlugatch,[2] Omer Blumen,[1] Keren Schwartzman,[1] Michal Salama,[1] Netanel Shpigel*, [1] and Daniel Sharon* [1]

1 Institute of Chemistry and the Center for Nanoscience and Nanotechnology, The Hebrew University of Jerusalem, Jerusalem, 919040, Israel

2 Department of Chemistry and BINA – BIU Center for Nanotechnology and Advanced Materials, Bar-Ilan University, Ramat-Gan 5290002, Israel

* Daniel Sharon – daniel.sharon@mail.huji.ac.il

* Netanel Shpigel- nshpigel@gmail.com

1. Materials

LiTFSI (99.95%), $MgCl_2$ (99.99%), $AlCl_3$ (99.999%), DME, THF, Diglyme, Triglyme, DiButhyl-magnesium, Phenylmagnesium chloride, and hexafluoroisopropanol were obtained from Sigma-Aldrich. MgTFSI2 (99.95%) was purchased from Solvonic. Mg $(BH_4)_2$ was obtained from ChemCruz. All Mg salts were dried under vacuum for 24 h prior to synthesis.

2. Synthesis of Mg-based solutions

All sample preparations and electrochemical measurements were carried out in an Ar-filled glovebox.

DCC: The Mg($AlCl_3$-nRnR′)2 electrolytes were prepared by reacting MgR_2 (for example, $MgBu_2$) and $AlCl_2R$ or $AlCl_3$ in hexane, followed by complete evaporation of the hexane and dissolution in the selected ether solvent.

APC: First, $AlCl_3$ was dissolved in dry THF solution in the desired concentration by very slow addition to the vigorously stirred solvent. Then, this solution was added dropwise to a predetermined quantity of 2 M Phenylmagnesiumchloride (PhMgCl) solution in THF. Both reactions are very exothermic. The resulting solution was stirred for an additional 16 h or more (at room temperature).

MACC: In a typical preparation of an electrochemically active MACC solution, we follow the following reaction: $2MgCl_2 + 1AlCl_3 \rightarrow Mg_2AlCl_7$, using $MgCl_2$ and $AlCl_3$ powders in tetrahydrofuran. Subsequently, stir and heat to ≥30.0 °C for several hours after which the solution may be returned to room temperature. The resulting solution is light yellow with no precipitation.

$MgTFSI_2$: predetermined amounts of $MgTFSI_2$ and $MgCl_2$ were added to DME/Diglyme/Triglyme (Dimethoxyethane) and stirred for 6 h at 70 ◦C.

$Mg[B(HFIP)_4]_2$: Mg $(BH_4)_2$ powder was dissolved in DME in a Schlenk flask inside the glovebox. $HOC(H)(CF_3)_2$ was slowly added into the stirred solution over a period of 1 h. After stirring at room temperature for 1 h, the flask was taken out of the glovebox and equipped with a Dimroth condenser, and the reaction was refluxed at 85 °C under argon for 2 h. After cooling down, the solvent was removed under vacuum. The resulting solid was further dried at room temperature for 24 h under high vacuum to remove excess fluorinated alcohol, solvent, and water residues. A 0.3 M of the resulted electrolyte was prepared in DME solution.

3. Electroanalytical Measurements

*Cell configuration and materials***.** All electrochemical measurements were performed in flooded 3 electrode glass cells. Mg strips with thickness of 100 μm were used as the counter, surface area of 3 cm^2, and reference

electrodes in all measurements. Before usage, the Mg foils were mechanically cleaned in the glovebox by scraping away the natural oxide coating with a glass blade. Cleaned Pt foils with surface area of 1 cm^2 were used as the working electrode for all metal deposition/dissolution measurements.

Electrochemical measurements: All sample preparations and electrochemical measurements were carried out in an Ar-filled glovebox (H_20/O_2 under 0.1 ppm) with a feedthrough connection to a multichannel VMP-2 portenitostat, Bio-Logic Co

Images of deposited Mg. HR-SEM imaging was performed using a Magellan XHR 400L FE-SEM (FEI Company).

4. Macrocycling profiles of Mg-based electrolyte solutions

Macrocycling measurements of the studied solutions at 25 % depths of discharge at different current densities, where Pt is used as WE and Mg foils as both CE and RE.

Figure S1. DCC based solutions

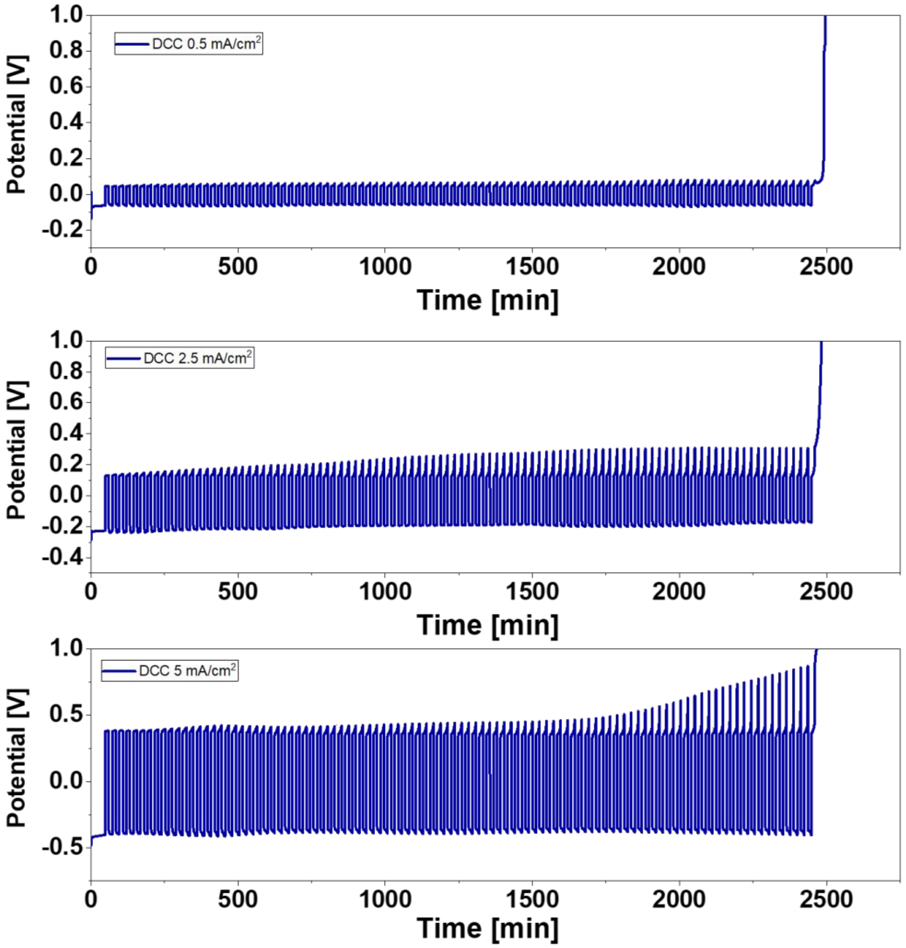

Figure S2. APC-based solutions

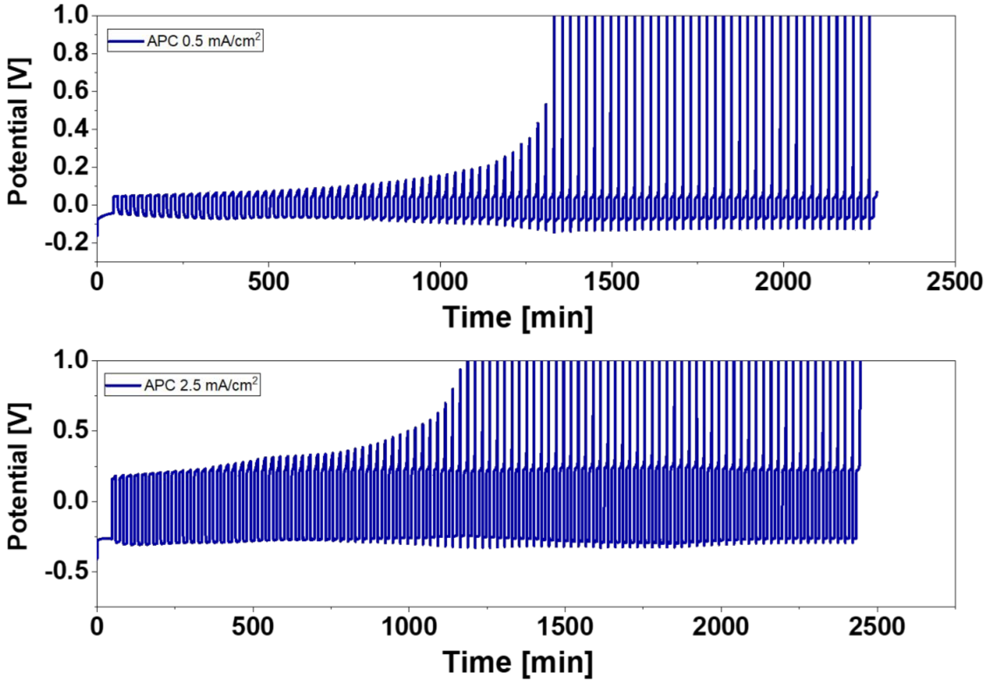

Figure S3. MACC-based solutions

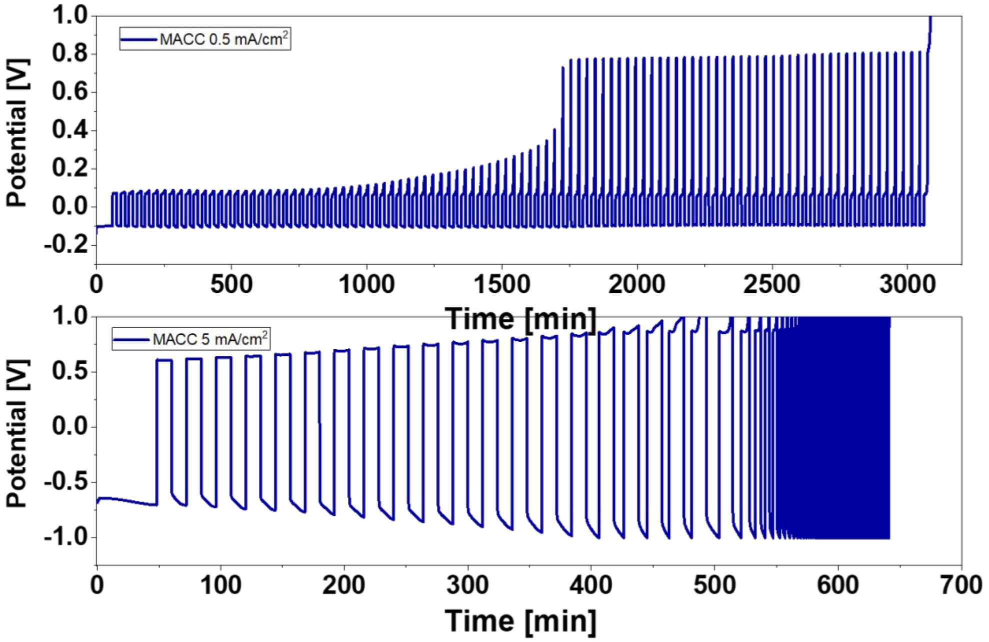

<u>Figure S4. $MgTFSI_2$-based solutions</u>

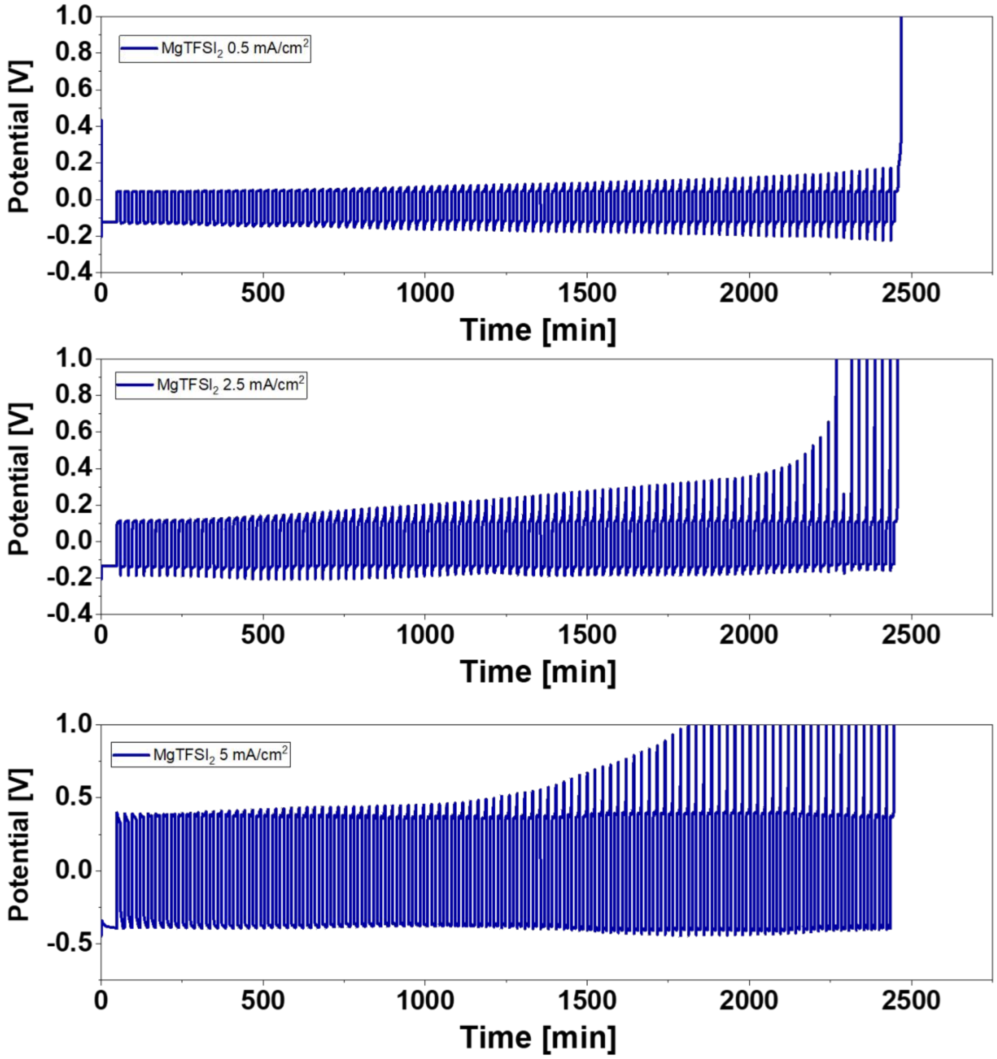

Figure S5. $Mg[B(HFIP)_4]_2$ -based solutions

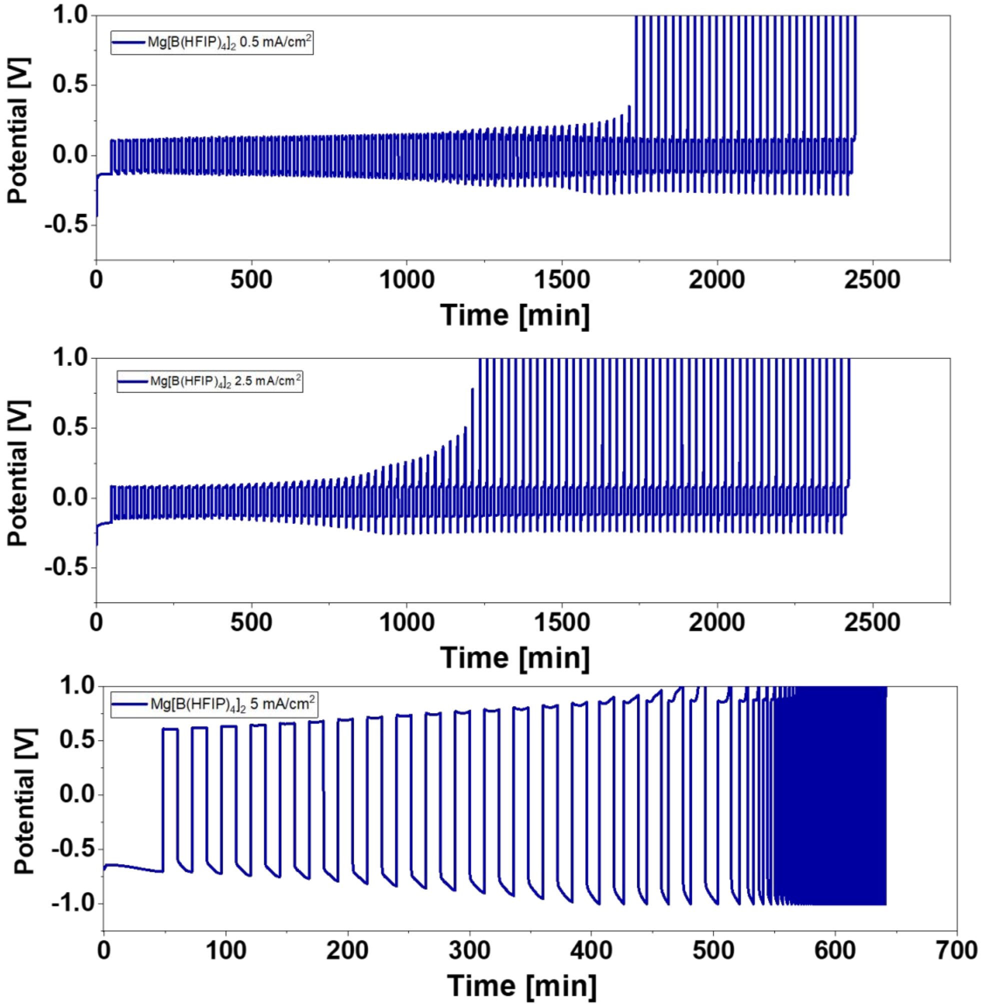

5. **Macrocycling profiles of Mg-based electrolyte solutions**

Macrocycling measurements of the studied solutions at 5 % depths of discharge at different current densities, where Pt is used as WE and Mg foils as both CE and RE.

<u>Figure S6. MACC-based solutions</u>

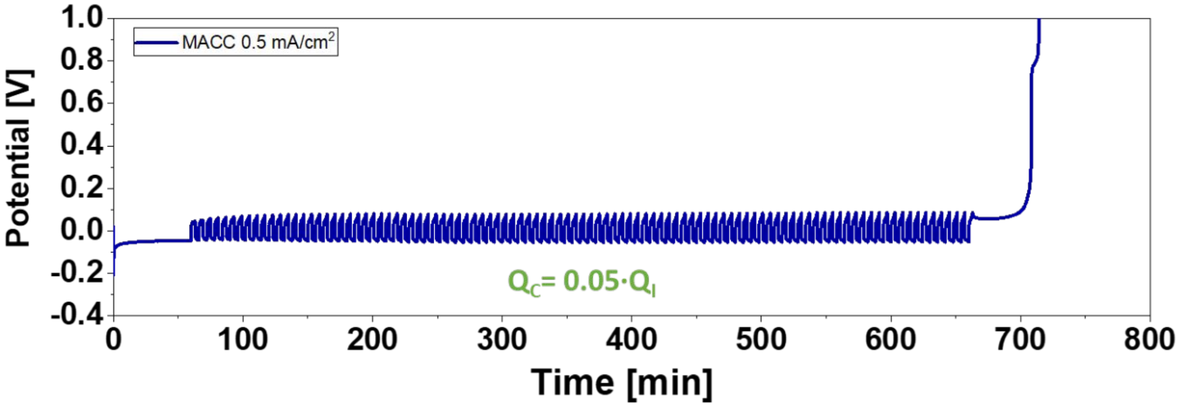

6. Motrpholohgy of electrodeposited Mg

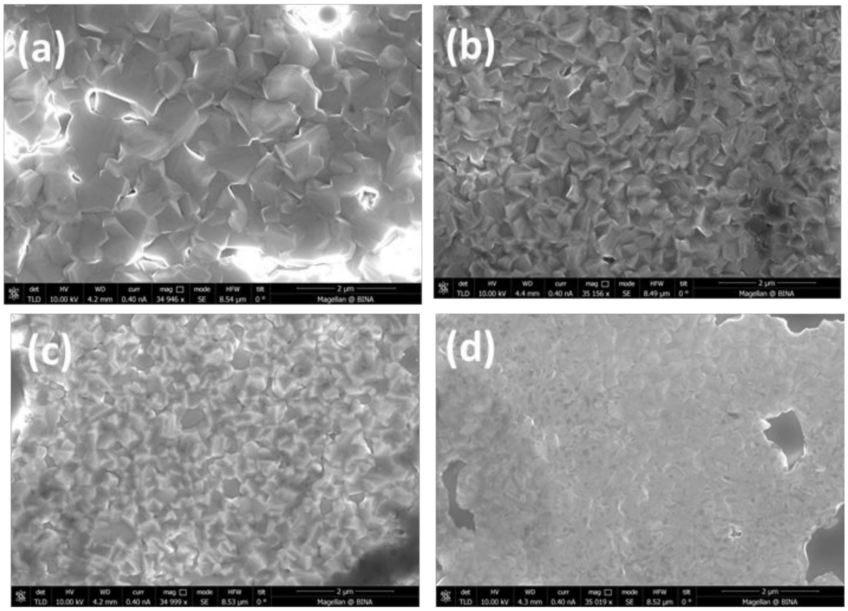

Figure S7. SEM images of Mg deposited on Pt electrode from DCC electrolyte solution on Pt electrodes at (a) 0.5, (b) 1, (c) 2.5 and (c) 5 mA/cm^2.

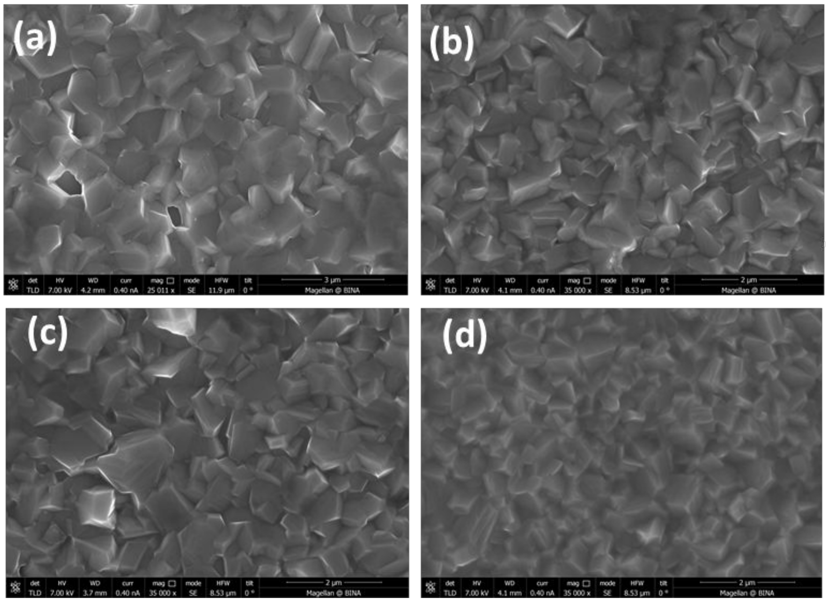

Figure S8. SEM images of Mg deposited on Pt electrode from APC electrolyte solution on Pt electrodes at (a) 0.5, (b) 1, (c) 2.5 and (c) 5 mA/cm^2.

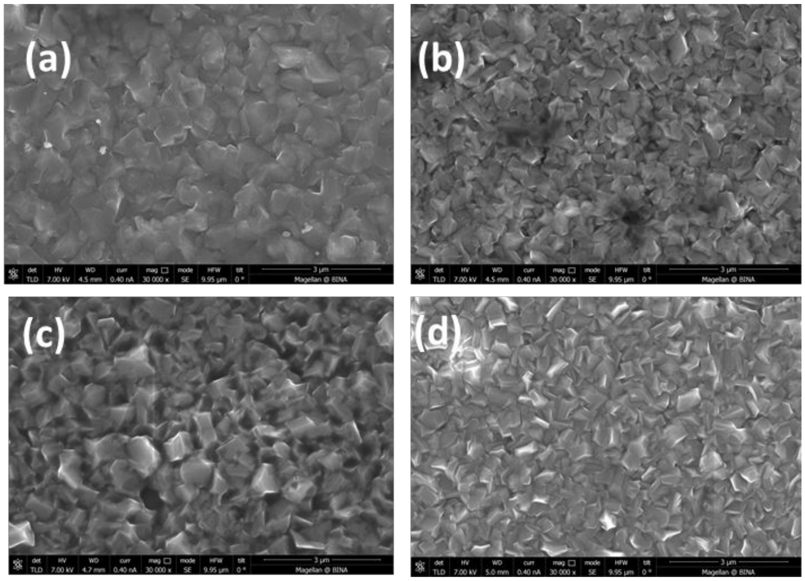

Figure S9. SEM images of Mg deposited on Pt electrode from MACC electrolyte solution on Pt electrodes at (a) 0.5, (b) 1, (c) 2.5 and (c) 5 mA/cm^2.

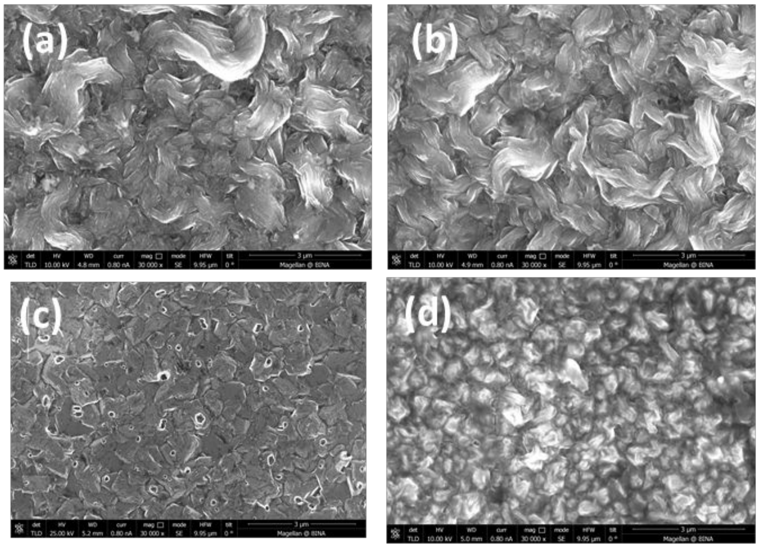

Figure S10. SEM images of Mg deposited on Pt electrode from $MgTFSI_2$-based electrolyte solution on Pt electrodes at (a) 0.5, (b) 1, (c) 2.5 and (c) 5 mA/cm^2.

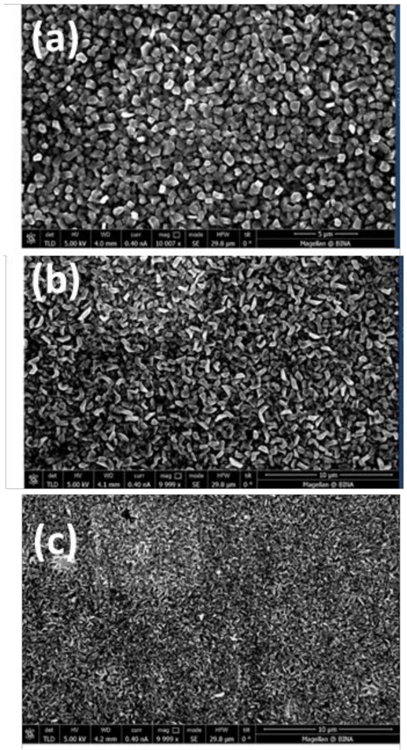

Figure S11. SEM images of Mg deposited on Pt electrode from a conditioned 0.3 M Mg[B(HFIP)4]2/DME electrolyte solution on Pt electrodes at (a) 0.5 (b) 1 and (c) 5 mA/cm^2.
